# Home modifications and disability outcomes: A longitudinal study of older adults living in England

**DOI:** 10.1016/j.lanepe.2022.100397

**Published:** 2022-05-04

**Authors:** Tarani Chandola, Patrick Rouxel

**Affiliations:** a Department of Sociology, University of Hong Kong, Jockey Club Tower, Pokfulam Road, Hong Kong; b School of Social Sciences, University of Manchester; c Faculty of Social Sciences, University of Hong Kong, Jockey Club Tower, Pokfulam Road, Hong Kong

**Keywords:** Housing, Disability, Ageing in place, Impairments, Falls, Pain, ICF model, Social participation, Home modifications, Home adaptations

## Abstract

**Background:**

There is limited evidence on the protective effect of housing modifications on disability outcomes among older adults. We examined whether external and internal housing modifications reduce the risk of a range of disability outcomes among older adults living in England.

**Methods:**

We analysed adults aged 60 and over from the English Longitudinal Study of Ageing, initially recruited in 2002/03. The longitudinal sample consisted of 32,126 repeated observations from 10,459 individuals across 6 waves with an average follow-up of 11·3 years. Participants were asked if their homes had external (widened doorways, ramps, automatic doors, parking and lift) and internal (rails, bathroom/kitchen modifications, chair lift) housing modifications. Mobility impairment was measured through reported difficulties in 10 activities including walking, climbing, getting up, reaching and lifting. Five disability outcomes were analysed (falls in the previous two years, pain, poor self-rated health, no social activities, and moving home within next two years) using two-way fixed effect models, controlling for key risk factors for disability.

**Findings:**

Greater mobility impairments increased the probability of falls, pain and poor self-rated health although this effect was significantly moderated by external housing modifications. Among older adults with severe mobility impairments, external housing modifications reduced the probability of falls by 3% (1%-6%), pain by 6% (4%-8%), and poor health by 4% (2%-5%). Moreover, external housing modifications reduced the probability of no social activities by 6% (5%-7%) and moving home by 4% (2%-5%) even among those without any mobility impairments. Internal housing modifications had similar, but less consistent effects on the disability outcomes.

**Interpretation:**

There was strong evidence that external housing modifications protected against a range of disability outcomes. Studies on reducing disability in ageing populations need to consider the role of housing modifications as key interventions to promote healthy ageing in place.

**Funding:**

Economic and Social Research Council ES/R008930/1 and ES/S012567/1

**Research in context**

**Evidence before the study**

We searched PubMed for articles published from 2000 to December 15, 2021, using the search terms (“aging/ageing” OR “older people/adults”) AND (“housing/home modifications/adaptations/interventions”) AND (“disability” OR “activities of daily living”, OR “mobility limitation/impairment”, OR “functional limitation/impairment”), with no language restrictions. Additionally, we reviewed all papers in Google Scholar that cited key systematic reviews and randomised controlled trials and longitudinal observational studies on home modifications to prevent disability outcomes.

There was strong and consistent evidence that home modifications as part of a multicomponent intervention reduced falls, but the use of multicomponent interventions makes it difficult to separate out the specific role of home modifications. Moreover, the relatively short follow-up periods of RCTs makes it hard to evaluate the impact of housing adaptations on social outcomes like ageing in place. There is very little comprehensive analysis of the effect of housing on disability. Housing modification effects on health and wellbeing, ageing processes, and social participation are less well evidenced. There are also significant gaps in knowledge on the effects of more major expenditure housing modifications such as providing showers or stair-lifts.

**Added value of the study**

This is the largest panel study on the role of housing modifications and disability outcomes in older adults, and with the longest follow-up period. There was strong evidence that installing external housing modifications (homes with widened doorways, ramps, automatic doors, parking and lift) reduced the incidence of falls by 3%. In addition, the study observed a range of health and social outcomes over 11·3 years on average, and found robust evidence that external modifications reduced the probability of pain by 6% and poor self-rated health by 4% among the mobility impaired, as well as reduced the incidence of no social activities by 6% and moving home by 4% among those without any mobility impairments.

**Implications of all the available evidence**

There is consistent evidence that housing modifications can prevent a range of disability outcomes ranging from functional (falls prevention) to health and wellbeing to social outcomes. Housing interventions should not be ignored in studies on disability and ageing.

Alt-text: Unlabelled box

## Introduction

For older adults with multiple, long-term conditions, accessible housing is key to their independence, safety and wellbeing.^1^ However, most of the housing stock in the UK is poorly designed for the rapidly ageing population,^2^ with only 7% of homes in England in 2014 meeting the minimum standard of accessibility.^3^

There is a surprising lack of research on housing and disability outcomes among older adults.^4^ Most housing interventions studies are concerned with falls, with much less convincing evidence on how housing modifications can improve quality of life and social outcomes.^5^ Moreover there is a reliance on evidence from multifactorial interventions in Randomised Control Trials (RCTs), which makes it hard to determine the specific effect of home modifications.^2^^,^^6^ This study examines the role of housing modifications in reducing falls and improving quality of life and social outcomes among older adults with and without disabilities.

Existing reviews of interventions to improve healthy ageing^7^ or risk factors for disability^8^ fail to mention any role for housing interventions. This is despite the importance of housing and environmental factors in conceptual models of disability like the International Classification of Functioning, Disability and Health (ICF) and the person-environmental fit models.^4^^,^^9^ In the ICF framework, disability is an umbrella term used to describe the combination of the negative aspects of functional loss, activity limitation, and participation restriction.^10^ Mobility impairment is the most prevalent form of disability facing older adults today.^4^ Disability is conceptualised as arising through the interaction of impairments with personal, social and environmental characteristics.^10^ Modifiable environmental features that enable participation in social roles/activities may be especially important in reducing disability outcomes.^9^ The disability outcomes framework^11^ measures meaningful outcomes from public policy strategies on improving areas of everyday life so that people with disability can achieve the same outcomes as people without disability. There are seven broad outcomes (including education, employment and financial security), but for purposes of this study, we focus on two- health and wellbeing and inclusive homes and communities. We focus on these two broad disability outcomes because there are limitations in the evidence in relation to housing modifications.^6^

Home modifications are structural changes in the indoor or immediate outdoor home environment to help people to be more independent and safer in their own home and reduce risk of injury.^12^ Modifications include changes to the structure of the dwelling (e.g. widening doors, or adding ramps), and the installation of assistive devices inside or outside the dwelling (e.g. grab rails, handrails, or lifts). Falls are the most common outcome examined in analyses of housing modifications.^6^ Multifactorial interventions in RCTs, where home modifications are just one among several interventions, can reduce the likelihood of falls and injury,^13^^,^^14^ and reduce fear of falling. However, in relation to other outcomes of disability, particularly related to pain, physical health and wellbeing, ageing at home and social participation, the evidence is more limited.^6^ External housing modifications may be especially important for the social activities of disabled adults (which requires external access) to enable them to be independent and have a social life, which in turn may impact on their health and wellbeing.

The evidence base for the effect of major expenditure home modifications such as installing showers or stair-lifts is limited.^15^ Moreover much of the research is unsystematic, with small sample sizes, making it difficult to make a compelling case for additional investment by policy makers.^16^ The RCT evidence is also problematic as the interventions are multifaceted, making it impossible to separate out the effect of home modifications alone. The short follow up periods of RCTs also means that more immediate functional outcomes are analysed (such as falls) rather than longer term social outcomes which are typically not observed within the RCT time frame.^6^

On the other hand, observational studies suffer from confounding and selection biases. Contrary to expectations, home modifications resulted in greater depressive symptoms as disability increased.^17^ In Wales, non-frail older adults with home modifications were more likely to become institutionalized than their peers without home modifications.^18^ This unexpected result may occur because people who receive a home modification are selected for a range of physical impairments. As impairment increases, the need for home adaptations increases^15^ to enable older adults with impairments to remain in their homes and allow for “ageing in place”.^6^ Housing modifications may not matter much for people without disabilities or with minimal disabilities. But they could make a profound difference in the quality of life and social outcomes for seriously disabled older adults through their greater susceptibility to environmental conditions.^19^

### Research Questions

1. Do home modifications reduce the risk of falls and improve the quality of life and social functioning among older adults?
2. Is the reduction in risk greater for more severely disabled older adults?

## Methods

### Study design and participants

The English Longitudinal Study of Ageing (ELSA) is a large scale longitudinal panel study of people aged 50 and over and their partners, living in private households in England.^20^ The initial sample was recruited in 2002/03 (wave 1) and has been refreshed at several waves, so not all respondents have participated since 2002. To date, 9 waves of ELSA are available for analyses with wave 9 survey conducted in 2018-2019 (each wave is conducted every 2 years).^21^

The ELSA w1-w9 merged individual level datasets included 90,074 observations from between 8,445 to 12,099 people surveyed at each wave (Table S1). However, not all the disability outcomes of interest were measured at each wave, resulting in a smaller pool of observations from waves 1,2,5,6,7, and 8. This resulted in 60,517 observations from 6 waves. Out of these, 32·1% had missing data on falls- as the questions were restricted to those aged 65 and over (60 and over in some waves). Out of the 41,111 observations from the 6 waves with no missing data on falls, 16·2% had missing data on moved home subsequently, which requires linked data from the next wave. Out of the 34,457 observations with no missing data on falls and moved home subsequently, there were 6·8% observations that had missing data on the covariates, with the bulk of the missingness was due to missing wealth data (6·1%). This resulted in an analytical sample size of 32,126 observations from 6 waves of ELSA data with a mean follow up time of 11·3 years (range 2-17 years).

**Variables (see Table S2)**

### Outcome assessment

A range of disability outcomes were analysed, ranging from functional to quality of life and social outcomes.

Falls: respondents were asked if they had fallen in the last two years (no=0, yes=1).

Pain: respondents were asked if they were often troubled with pain (no=0, yes=1).

Poor health: respondents were asked if their health was excellent/very good (coded as 0) or good/fair/poor (coded as 1).

No social activities: respondents were asked if they were currently participating in any of the following social activities: in paid work/self-employment/voluntary work/education/caring/looking after the home or family (coded as 0 if not participating in any activities and 1 if any of these activities).

Moved home: respondents were coded as having moved home by the next wave if their address had changed at the subsequent ELSA wave (around 2 years later).

### Exposure assessment

Housing modifications: Respondents were asked if their home had any of the following: widened doorways or hallways/ramps or street level entrances /handrails/automatic or easy open doors/accessible parking or drop off site/bathroom modifications/kitchen modifications/lift/chair lift or stair glide/alerting devices/any other special features. We carried out a series of polychoric correlations at each wave and found consistent evidence for a two-factor solution that combined (1) widened doorways, ramps, automatic doors, parking and lift (which we labelled external modifications for access) and (2) rails, bathroom/kitchen modifications, chair lift (which we labelled internal modifications). We created binary variables for external and internal modifications if respondents lived in homes with any of those features.

Impairment: Following the ICF framework, mobility impairment was our key measure of disability. We used the ELSA questions on mobility limitations: if the respondent had long term difficulty walking 100 yards/sitting for about two hours/getting up from a chair after sitting for long periods/climbing one flight of stairs without resting/stooping, kneeling, or crouching/reaching or extending your arms above shoulder level/pulling or pushing large objects like a living room chair /lifting or carrying weights over 10 pounds, like a heavy bag/picking up a 5p coin from a table. We created a summary score ranging from 0-10 adding up each item.

### Covariates

Covariates were controlled for in the analyses to examine whether housing modifications influenced the outcomes independent of key factors associated with disability. The ICF framework includes health conditions and impairments in body structures and functions. Chronic health conditions were measured through self-reports of the following- high blood pressure, diabetes, cancer, lung disease, heart conditions, stroke, psychological conditions, arthritis, cataract, Parkinson's, osteoporosis, Alzheimer's, dementia, and memory related problems. Sight problems: respondents were asked if their eyesight was excellent/very good (coded as 0) or good/fair/poor/blind (coded as 1)). Similarly, hearing problems were coded from asking if their hearing was excellent/very good (coded as 0) or /good/fair/poor (coded as 1). The 8 item CES-D questionnaire was used with a cut off at 3 or more indicating depressive symptoms.^22^ A combined summary score from 6 Activities of Daily Living (ADL) questions was used to measure functional difficulties. Socio-demographic characteristics were included as disabled adults are more likely to live in poverty. These included living in single vs multiple person households, being in a coupled relationship (vs not in a coupled relationship), and household wealth quintiles. Exercise was included as it is a key modifiable factor for improving disability outcomes. Participants reported their frequency in participating in moderate sports or activities as more than once/week, once/week, one-three times/month or hardly/never.

## Statistical analysis

We estimated two-way fixed effects models in STATA14, which is a common method for estimating causal effects from panel data.^23^ These models adjust for unobserved unit (person)-specific and time (wave)-specific confounders at the same time. Interaction terms between mobility impairments and housing modifications were estimated to answer RQ2. Linear regression fixed-effect models rather than logistic regression models were estimated for the main analyses, even though each of the outcome variables were binary. This is because it is not possible to derive meaningful predicted probabilities from logistic fixed-effects models which assume that the fixed effects are zero. Therefore, we used linear regression models to estimate the associations with the binary dependent outcome variables and used the STATA *margins* command to estimate the predicted levels of the dependent variable by different categories and combinations of the explanatory variables. We interpreted these predicted levels as percentages (due to the binary nature of the dependent variables).

Missing data: All available cases were analysed for the main analyses with ELSA longitudinal weights at wave 9 applied for sensitivity analyses. Table S1 describes how the main analytical sample was derived from the different waves of ELSA. The majority of missingness was by design, as some ELSA waves did not include the variables of interest or respondents were not eligible due to their age. We assume that these data are missing completely at random. Some missingness was due to loss to follow up (16.2%), and missing wealth data (6.1%). The derived ELSA longitudinal weights (which assume that these data are missing at random) correct for both sampling variation (taking into account different probabilities of being selected into the ELSA sample at different weights) as well as non-response and attrition between waves and make the analyses representative of the older population in England.^24^ Inverse probability weighting methods are sometimes preferred over multiple imputation methods to take account of missing data in longitudinal surveys with missing values on several rather than just one or two variables.^25^ ELSA longitudinal weights are only available for core sample members who have participated in every wave of ELSA, so applying these longitudinal weights results in a marked reduction in observations from 32,126 to 14,185 (Table S1). This 56% drop in the analytical sample size is a major problem that results in reduced statistical power. Consequently, for the main analyses, we used the unweighted sample of 32,126 observations. Supplementary analyses were carried out using the longitudinal weights, comparing estimates from the unweighted and weighted analyses.

### Role of the funding source

The funder of the study had no role in study design, data collection, data analysis, data interpretation, or writing of the report.

## Results

The prevalence of disability outcomes was higher among older adults with mobility impairments and housing modifications (either external or internal modifications), except for the outcome of “no social activities” (Table 1). Unsurprisingly, the rates of ADL limitations and chronic health problems were higher among those with mobility impairments. Home modifications (either external or internal) were much higher among the poorest wealth quintile in the sample, compared to the wealthiest quintile. The unweighted distribution of the variables in Table 1 was remarkably similar to the weighted distribution (Table S3).

**Table 1:** Percentage of disability outcome observations (falls, poor health, pain, no social activities and moved home) and key covariates by exposure variables (mobility impairments and external/internal housing modifications): ELSA analytical sample (N=32,126).

	No mobility impairments		1+ mobility impairments		No mobility impairments		1+ mobility impairments		
	No extmods	External mods	No ext mods	External mods	No int mods	Internal mods	No int mods	Internal mods	*n (out of 32126)*
Falls	18.9%	21.6%	35.3%	38.4%	18.9%	22.1%	`31.6%	44.1%	*9,317*
Poor health	8.4%	8.6%	39.2%	42.6%	7.9%	11.9%	33.3%	52.4%	*8,594*
Pain	17.2%	18.1%	56.6%	63.1%	17.0%	19.5%	54.2%	64.8%	*13,152*
No social activities	29.8%	21.1%	40.8%	40.1%	28.5%	28.5%	35.8%	49.9%	*11,425*
Moved home	4.4%	5.3%	5.1%	6.9%	4.6%	4.5%	5.3%	5.7%	*1,629*
1+ ADL	4.0%	4.1%	43.6%	50.9%	3.5%	7.3%	35.1%	63.7%	*8,950*
1+ health condition	72.0%	76.6%	91.3%	94.7%	72.0%	77.5%	90.0%	95.6%	*26,959*
Poor sight	42.5%	42.5%	59.6%	61.3%	41.9%	46.3%	56.2%	66.9%	*16,916*
Poor hearing	50.3%	49.1%	62.4%	62.8%	49.7%	53.0%	60.9%	65.4%	*18,405*
Dep sympt	10.1%	8.5%	27.6%	30.5%	9.5%	12.6%	23.2%	37.5%	*6,590*
Single household	24.4%	23.0%	33.5%	36.1%	23.7%	27.1%	29.5%	42.6%	*9,607*
Couple Relationship	68.7%	71.7%	58.7%	57.3%	69.7%	65.3%	63.0%	49.8%	*20,211*
No moderate activities	6.5%	4.6%	26.9%	34.0%	5.8%	8.8%	19.7%	44.4%	*6,116*
Poorest quintile	9.8%	8.9%	20.1%	26.5%	8.4%	17.8%	16.7%	30.1%	*5,283*
Wealthiest quintile	29.2%	37.6%	16.3%	17.7%	31.3%	25.3%	19.9%	10.3%	*7,194*

**Abbreviations:**Ext: external; Int: internal; Mods: modification; ADL: Activities of Daily Living; Dep sympt: Depressive symptoms.

**Figure 1:**
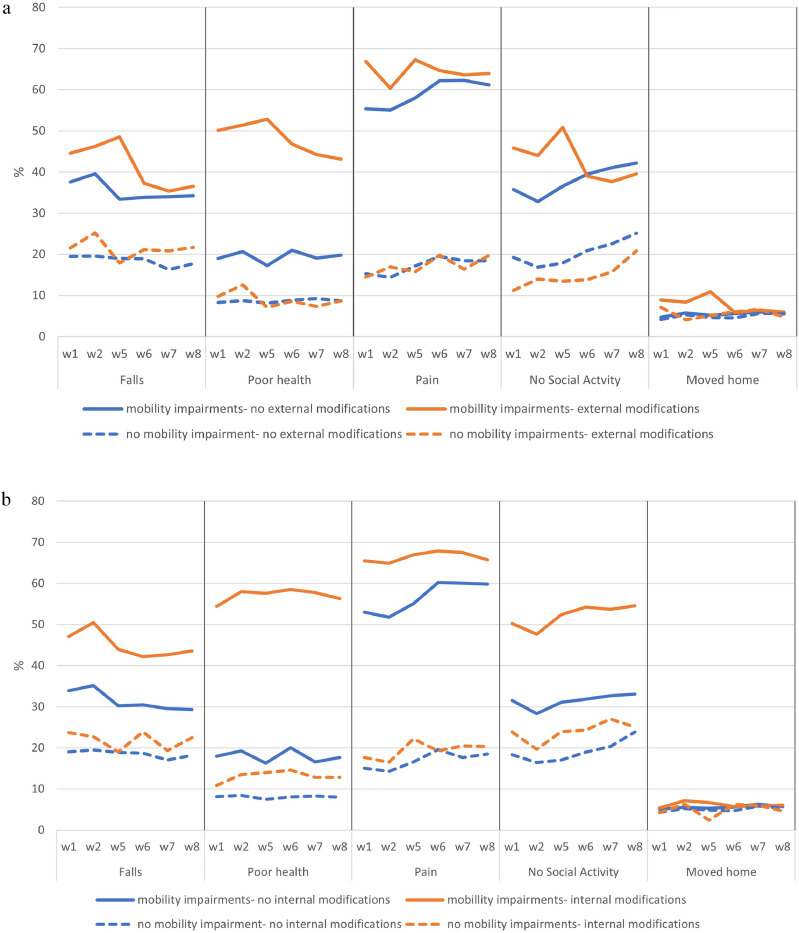
(1a) Percentage distribution of disability outcomes by ELSA wave (w) and external modifications and mobility impairments; (1b) ercentage distribution of disability outcomes by ELSA wave and internal modifications and mobility impairments.

The change in the prevalence of the five disability outcomes across ELSA waves and by mobility impairment and housing modifications are shown in Figures 1a (for *external* housing modifications) and 1b (for *internal* housing modifications). There was a clear pattern of higher levels of disability outcomes for ELSA respondents with mobility impairments (the solid lines) compared to those without any mobility impairments (the dotted lines). Moreover, among those with at least 1 mobility impairment, the gap between those with and without external modifications in earlier waves appeared to decrease by the later waves of ELSA (Figure 1a). However, there was no such reduction in the prevalence of disability outcomes for ELSA respondents with mobility impairments and *internal* modifications at later waves (Figure 1b). These figures show cross-sectional associations (prevalence). To examine within person changes in impairment, housing modifications, and disability outcomes, we examined fixed effect models.

**Figure 2:**
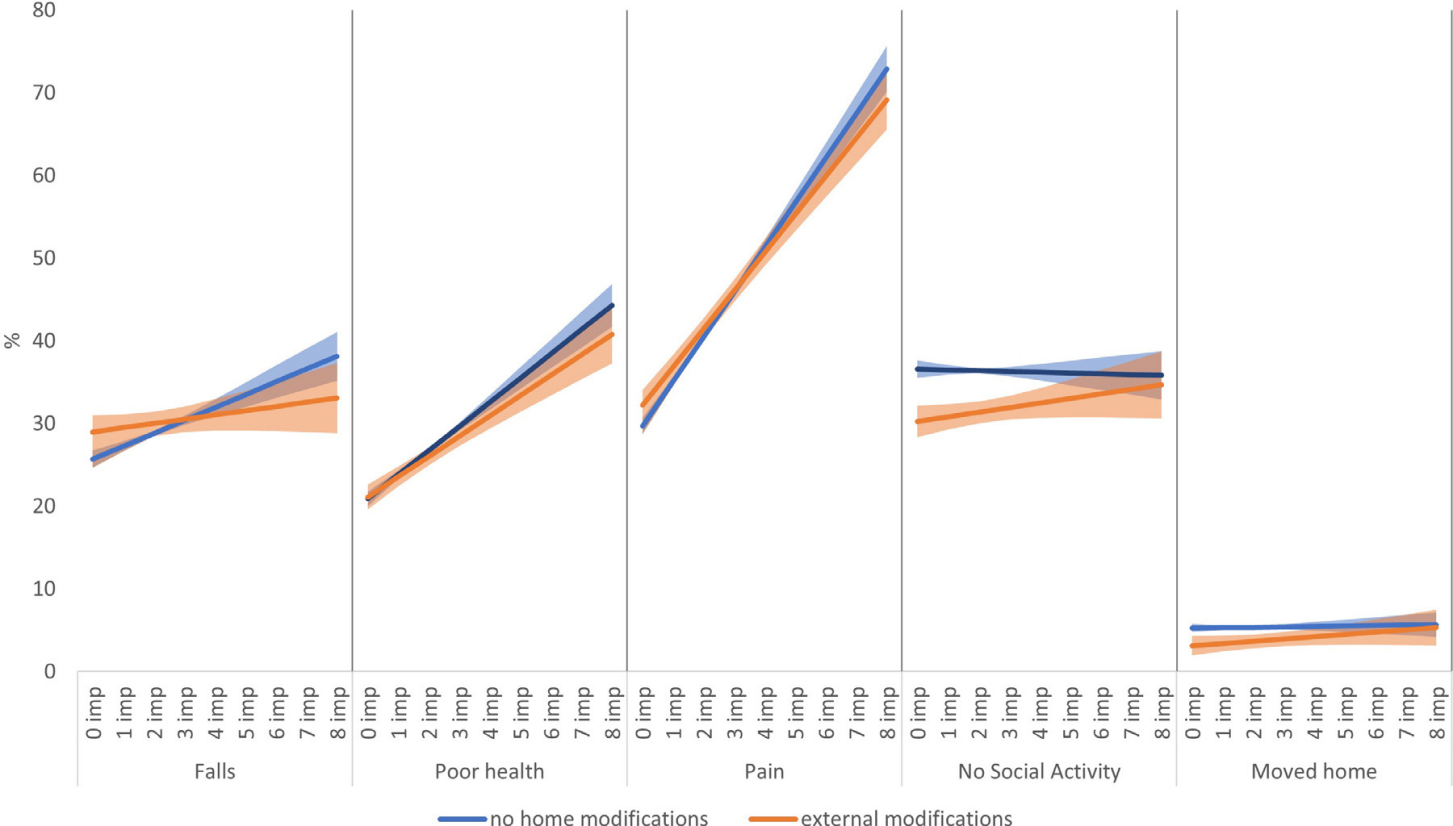
Predicted probability of falls, poor health, pain, no social activities and moving home by mobility impairments and external housing modifications.

The regression coefficients from the two-way fixed effects models on the five disability outcomes with external housing modifications are shown in Tables S4 (full model with all the coefficients) and Table 2a (selected model coefficients only). The within-person goodness of fit (R-sq) for each outcome ranged from 8% for poor health to less than 1% for moved home. The incidence of disability outcomes was- falls (22·2%), pain (21·8%), poor self-rated health (13·9%), no social activities (25·1%) and moving home (4·5%). An increase in one mobility impairment resulted in an increase in the probability of falls by 0·01 (or 1%). External home modifications increased the risk of falls by 1%, although not significantly (Table 2a, M1: Falls). There was evidence of a significant negative interaction between mobility impairments and external modifications (Table 2a, M2: Falls). As people became more mobility impaired, the probability of falls decreased by 1% among those who had their homes modified for external access. This interaction effect is shown in Figure 2 (falls) where among those with no impairments, the probability of falls among those with external modifications is slightly higher than those without such housing modifications. But as people became more mobility impaired, external modifications protected them from the risk of falling so that among people with 8 mobility impairments, the probability of falling decreased by 3% (CI: -0.06 to -0.01) when compared to those with the same level of impairments but living in homes without any external modifications (see Table 3a: Falls).

**Table 2:** Selected regression coefficients (standard errors) from two-way fixed effects models of disability outcomes.

**2a:** Models with **external** housing modifications (full model coefficients in Table S4)			
	**Models with external housing modifications**		
*Outcome variables*	**Mob imp (range:0-10)**	**Ext mods (ref: no ext mods)**	Mob imp*Ext mods
Falls (M1)	**0·014 (0·002)**	0·009 (0·009)	..
Falls (M2)	**0·016 (0·003)**	**0·033 (0·011)**	**-0·010 (0·003)**
Poor health (M1)	**0·028 (0·002)**	-0·009 (0·007)	..
Poor health (M2)	**0·029 (0·002)**	0·002 (0·008)	**-0·005 (0·002)**
Pain (M1)	**0·052 (0·002)**	0.007 (0·008)	..
Pain (M2)	**0·054 (0·002)**	**0·025 (0·010)**	**-0·008 (0·002)**
No Social Act. (M1)	0.0003 (0·002)	**-0·048 (0·008)**	..
No Social Act. (M2)	-0·001 (0·002)	**-0·063 (0·010)**	**0·006 (0·003)**
Moved home (M1)	**0·002 (0·001)**	**-0·028 (0·005)**	..
Moved home (M2)	0·001 (0·001)	**-0·035 (0·006)**	0·003 (0·002)
**2b:** Models with **internal** housing modifications (full model coefficients in Table S5)			
	**Models with internal housing modifications**		
*Outcome variables*	**Mob imp (range:0-10)**	**Int mods (ref no int mods)**	Mob imp*Int mods

Falls (M1)	**0·013 (0·002)**	0·013 (0·009)	..
Falls (M2)	**0·014 (0·003)**	0·015 (0·012)	-0·001 (0·003)
Poor health (M1)	**0·028 (0·002)**	0·014 (0·007)	..
Poor health (M2)	**0·029 (0·002)**	**0·024 (0·009)**	**-0·004 (0·002)**
Pain (M1)	**0·053 (0·002)**	-0·009 (0·008)	..
Pain (M2)	**0·058 (0·003)**	**0·027 (0·011)**	**-0·013 (0·002)**
No Social Act. (M1)	0·001 (0·002)	**-0·025 (0·009)**	..
No Social Act. (M2)	-0·002 (0·003)	**-0·046 (0·012)**	**0·008 (0·003)**
Moved home (M1)	0·002 (0·001)	-0·008 (0·005)	..
Moved home (M2)	-0·0003 (0·001)	**-0·022 (0·006)**	**0·005 (0·002)**

**Bold** coefficients denote statistical significance at p<0.05

All Models control for:

Mobility impairment, external/internal housing modifications, Activities of Daily Living difficulties, health conditions, sight and hearing problems, depressive symptoms, moderate physical activity, household size, coupled relationship, wealth quintiles and wave. In addition, the models include the 4 disability outcome measures as control variables out of the five disability outcomes: falls, pain, poor health, no social activities, moved home,

M1: Model 1 without interaction between mobility impairment and external/internal housing modifications

M2: Model 2 with interaction between mobility impairment and external/internal housing modifications (Mob imp*Ext/Int mods)

Abbreviations:

Mob: mobility; Imp: impairment; Ext: external; Int: internal; Mods: modification; Act: activities.

**Table 3:** Predicted probabilities (95% CI) of disability outcomes estimated from the interaction of mobility impairments and external/internal housing modifications (from Tables S4 and S5).

**3a:** Difference in predicted probabilities of living in a house with **external** modifications (compared to no external modifications) across levels of mobility impairments					
Mobility impairments	Falls	Poor health	Pain	No Social Act	Moved home
none	0·03 (0·01 to 0·05)	0·01 (-0·002 to 0·02)	0·02 (0·01 to 0·03)	-0·06 (-0·07 to -0·05)	-0·04 (-0·05 to -0·02)
1	0·02 (0·01 to 0·04)	0·003 (-0·01 to 0·01)	0·01 (0·002 to 0·02)	-0·05 (-0·07 to -0·04)	-0·03 (-0·04 to -0·02)
2	0·01 (0·001 to 0·03)	-0·003 (-0·01 to 0·01)	0·002 (-0·01 to 0·01)	-0·05 (-0·06 to -0·04)	-0·03 (-0·04 to -0·02)
3	0·01 (-0·01 to 0·02)	-0·01 (-0·02 to 0·001)	-0·01 (-0·02 to 0·003)	-0·05 (-0·06 to -0·04)	-0·03 (-0·04 to -0·02)
4	-0·002 (-0·02 to 0·01)	-0·01 (-0·02 to -0·004)	-0·02 (-0·03 to -0·01)	-0·04 (-0·05 to -0·03)	-0·02 (-0·04 to -0·01)
5	-0·01 (-0·03 to 0·01)	-0·02 (-0·03 to -0·01)	-0·03 (-0·04 to -0·01)	-0·04 (-0·05 to -0·02)	-0·02 (-0·04 to -0·005)
6	-0·02 (-0·04 to 0·001)	-0·02 (-0·04 to -0·01)	-0·04 (-0·05 to -0·02)	-0·03 (-0·05 to -0·02)	-0·02 (-0·04 to 0·001)
7	-0·03 (-0·05 to 0-0·003)	-0·03 (-0·05 to -0·01)	-0·05 (-0·06 to -0·03)	-0·03 (-0·05 to -0·01)	-0·01 (-0·04 to 0·01)
8	-0·03 (-0·06 to -0·01)	-0·04 (-0·05 to -0·02)	-0·06 (-0·08 to -0·04)	-0·02 (-0·04 to -0·003)	-0·01 (-0·04 to 0·01)

**3b:** Difference in predicted probabilities of living in a house with **internal** modifications (compared to no internal modifications) across levels of mobility impairments					

Mobility impairments	Falls	Poor health	Pain	No Social Act	Moved home

none	0·01 (-0·01 to 0·04)	0·02 (0·01 to 0·04)	0·03 (0·01 to 0·05)	-0·05 (-0·07 to -0·02)	-0·02 (-0·03 to -0·01)
1	0·01 (-0·01 to 0·03)	0·02 (0·01 to 0·04)	0·01 (-0·004 to 0·03)	-0·04 (-0·06 to -0·02)	-0·02 (-0·03 to -0·01)
2	0·01 (-0·005 to 0·03)	0·02 (0·002 to 0·03)	0·001 (-0·02 to 0·02)	-0·03 (-0·05 to -0·01)	-0·01 (-0·02 to -0·002)
3	0·01 (-0·01 to 0·03)	0·01 (-0·002 to 0·03)	-0·01 (-0·03 to 0·003)	-0·02 (-0·04 to -0·01)	-0·01 (-0·02 to 0·003)
4	0·01 (-0·01 to 0·03)	0·01 (-0·01 to 0·03)	-0·03 (-0·04 to -0·01)	-0·02 (-0·03 to 0·004)	-0·001 (-0·01 to 0·01)
5	0·01 (-0·01 to 0·03)	0·01 (-0·01 to 0·02)	-0·04 (-0·06 to -0·02)	-0·01 (-0·03 to 0·01)	0·004 (-0·01 to 0·02)
6	0·01 (-0·02 to 0·04)	0·001 (-0·02 to 0·02)	-0·05 (-0·07 to -0·03)	<0·001 (-0·03 to 0·03)	0·01 (-0·01 to 0·02)
7	0·01 (-0·02 to 0·04)	-0·002 (-0·03 to 0·02)	-0·07 (-0·09 to -0·04)	0·01 (-0·02 to 0·04)	0·01 (-0·004 to 0·03)
8	0·01 (-0·03 to 0·05)	-0·01 (-0·04 to 0·03)	-0·08 (-0·11 to -0·05)	0·02 (-0·02 to 0·05)	0·02 (-0·001 to 0·04)

Abbreviations: Social Act: Social Activities.

In terms of the risk of poor self-rated health and pain conditions, there was a similar pattern where among those with no mobility impairments, there was a slightly raised probability of these disability outcomes when comparing those living with external modifications to those without (Figure 2: poor health and pain). However, as people became more mobility impaired, the probability of poor self-rated health decreased among those who modified their homes for external access. The risk of poor health decreased by 4% (CI: -0.05 to -0.02) and the risk of pain by 6% (CI: -0·08 to 0·04) among those with 8 mobility impairments and external modifications compared to those with the same level of impairment but without any external home modifications (Table 3a: poor health and pain).

In terms of the risk of not participating in any social activities and moving home within 2 years, the main effect of external modifications was negative (Table 2a, M1: no social activity and moved home). The predicted graph including the interaction effect between mobility impairment and external modifications (Figure 2: no social activity) shows that among those with no mobility impairments and external modifications, the probability of not participating in any social activities was around 6% (CI: -0·07 to -0·05) lower compared to those living in homes without external modifications (Table 3a: no social activity). This protective effect of external modifications tended to reduce as mobility impairments increased, but even among those with 8 mobility impairments, the probability of not participating in any social activities was lower among those with external modifications compared to those without (Figure 2: moved home). A similar protective pattern was found for moving in the next two years where the probability was around 2% (CI: -0·03 to -0·01) lower for those with no mobility impairments living in homes with external modifications (Table 3a: moved home). Moreover, there was no evidence any interaction between mobility impairments and external modifications (Table 2a, M2: moved home).

**Figure 3:**
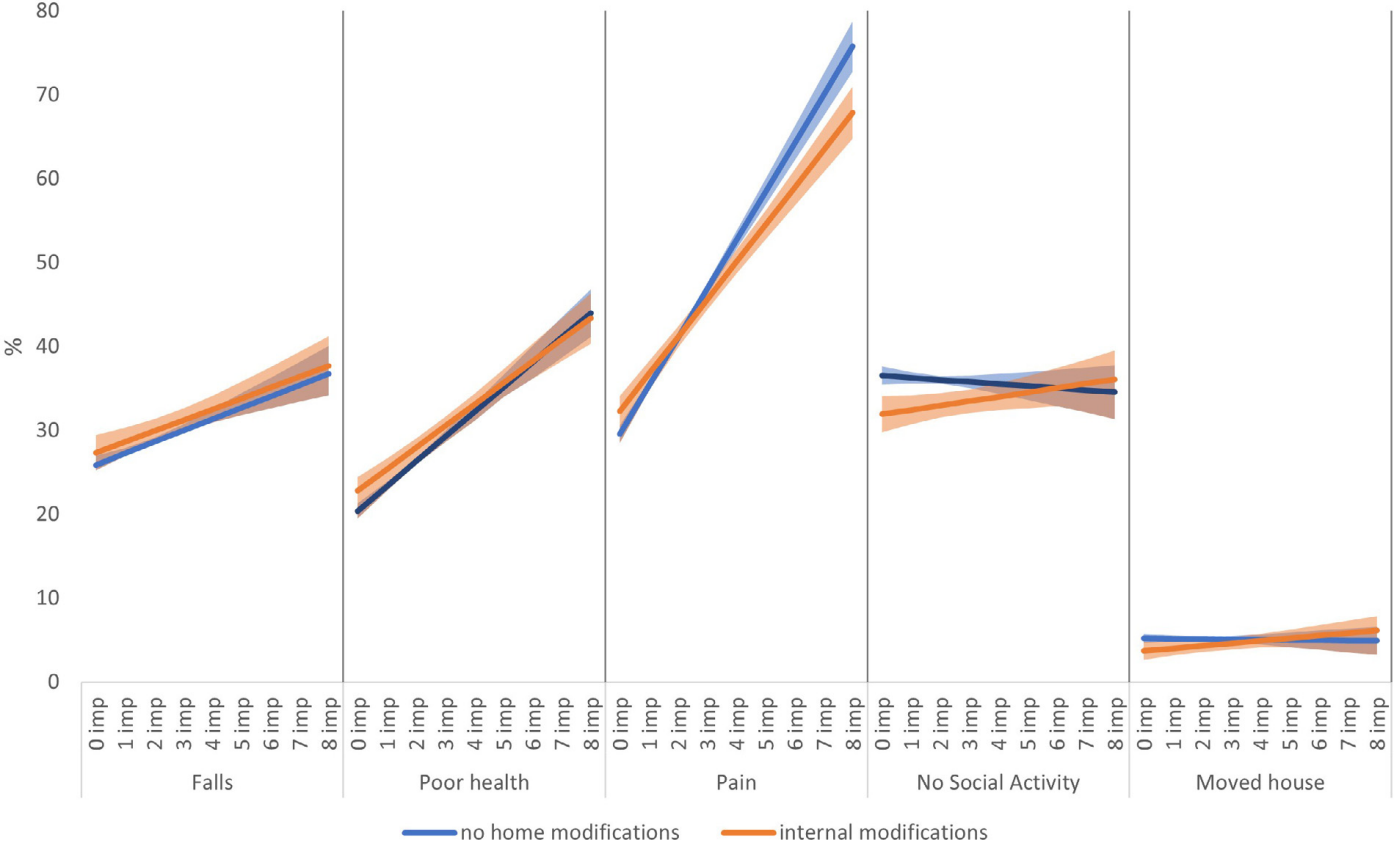
Predicted probability of falls, poor health, pain, no social activities and moving home by mobility impairments and external housing modifications.

There was some evidence for a similar protective effect of internal home modifications on the probability of most of the disability outcomes except for falls (selected coefficients in Table 2b; full model with all the coefficients in Table S5). Internal modifications reduced the probability of pain among those with greater mobility impairments (compared to those without any mobility impairments- see Figure 3: pain). Older adults with no mobility impairments living in homes with internal modifications tended to develop poorer health than their peers living in homes without internal modifications. As mobility impairments increased, this gap reduced (Figure 3: poor health). In terms of the risk of not participating in any social activities (Figure 3: no social activity) and moving home (Figure 3: moved home), internal modifications protected older people with few mobility impairments from these disability outcomes. However, as mobility impairments increased, the protection offered by such internal modifications decreased.

We conducted two types of sensitivity analyses. We estimated the same models using longitudinal weights for the reduced sample of core members who had participated in every wave of ELSA since wave 1 (Table S6). We obtained very similar estimates of the fixed effects in the weighted analyses to the unweighted analyses, although in some instances the coefficients were no longer statistically significant, probably to the very large reduction in the sample size. We also estimated fixed effect logistic regression models (with and without the longitudinal weights) where similar patterns in terms of statistical significance were observed to the linear regression models (Table S6). For some of the models, when comparing the weighted and unweighted coefficients, the size of the coefficients changed although with this may be expected as interaction coefficients were presented. However, there was a consistent pattern of much larger standard errors for coefficients from the weighted models because of the much smaller sample with longitudinal weights.

We tested the strict exogeneity assumption which rules out feedback from the dependent variables to the explanatory variables. The weighted fixed effects models in Table S6 were re-estimated, this time including the explanatory variables lagged forward by one wave. The null hypothesis, that these lagged explanatory variables are strictly exogenous, was tested by examining the joint significance F-test of all the lagged variables. For each of the disability outcomes, the significance of the F-test was >0.05, suggesting that there is no feedback observed and that the strict exogeneity assumption held.

## Discussion

We found consistent evidence that external housing modifications (widened doorways, ramps, automatic doors, parking and lift) helped to reduce a range of disability outcomes- falls, poor health, and pain conditions for older adults with significant mobility impairments. Moreover, external home modifications reduced the probability of not participating in any social activities and moving home at all levels of mobility impairments. A similar protective effect of internal housing modifications was observed for pain, no social activities, and moving home, but not in relation to falls or poor health.

This research specifically addressed the lack of comprehensive analysis on the effect of housing on disability.^26^^,^^6^ The short follow up periods of RCTs make analysis of longer term social outcomes problematic,^27^ which is why we used a longitudinal study with a long follow up period. Similar to existing research, we found evidence that (external) housing modifications reduced the probability of falls. However, it is difficult to compare the effect size from this study to intervention effects from RCTs as the interventions are multifactorial. We confirmed results from existing qualitative studies on the effect of housing modifications on improving quality of life and reducing pain.^6^ We also added to the very limited research on the role of housing interventions on enabling social activities and ageing in place.^6^ Moreover, the effect sizes of the coefficients of housing modifications and the interaction terms with mobility impairments (between 1% to 6%) is comparable to the effect size of transitioning from the wealthiest to the poorest groups (0.02% to 6%).

The predicted probabilities from these interaction effects all suggest some degree of protection from disability outcomes from external housing modifications, particularly among the most mobility impaired for the outcomes of falls, pain and health. This pattern is in line with results from previous research which found as frailty increased, home modifications reduced the chances of moving to an institution.^18^

The key message of this research is that despite the very consistent role of housing factors in influencing and moderating disability outcomes in older adults, there is woeful lack of consideration of housing factors in ageing^7^ and disability^8^ research. This is further exemplified by the lack of housing modifications data collected in many longitudinal studies of ageing. For example, questions about “ramp access” in homes were only asked in 6 out of the 18 studies in the Global Gateway for Ageing (available at https://g2aging.org/) and about “widened doorways” in only 3 of the studies.

While we found some evidence for the strict exogeneity assumption, there may be other sources of bias and confounding. There was a considerable drop in the sample size when the longitudinal weights were applied. While this makes the weighted analyses representative of the population aged 60 and over living in private households in England, our main set of analyses was based on the unweighted models. As the coefficients from the unweighted and weighted models for the independent variables and their interactions are similar for the weighted and unweighted models, the bias arising from missing data and selective attrition is mitigated to some extent. We also used a linear regression fixed effect model to analyse binary outcomes, because the predicted probabilities from a logistic fixed effect model are hard to interpret.^28^ This means that the estimates of the probabilities derived from the linear fixed effect models may not be robust, although in sensitivity analyses, the direction and statistical significance of the estimates from the linear and logistic models were the same. The plausibility of the predicted probabilities of the binary disability outcomes from the linear regression models was examined by comparing them with the range of probabilities of the disability outcomes among those with and without mobility impairments and housing modifications, described in Figures 1a and 1b. The predicted probabilities were within the range of probabilities from the descriptive statistics, suggesting that the estimates from the linear regression models were not estimating implausible values outside the binary range of the probabilities. All the data were self-reported by ELSA respondents, which may result in measurement bias. However, by analysing within person changes in the fixed effects model, time constant biases in such self-reports are eliminated.

This study found consistent evidence that external housing modifications can reduce a diverse range of disability outcomes, using high quality longitudinal data on a representative sample of the older adult population living in England. This adds considerably to the lack of evidence in relation to housing, ageing and disability outcomes, particularly in the UK context. Housing interventions can reduce disability outcomes and should not be ignored in studies on disability and ageing.

## Contributors

TC and PR contributed to the methods, variable construction, statistical analysis, and writing of the original and final drafts. PR conducted and wrote the literature review. TC contributed to the conceptualisation of the study. Both authors had full access to all the data and accept the final responsibility to submit for publication.

## Data sharing

Data from the English Longitudinal Study of Ageing (DOI: 10.5255/UKDA-SN-5050-24) are available to download from the UK Data Service (https://beta.ukdataservice.ac.uk/datacatalogue/studies/study?id=5050). The STATA ‘do’ file detailing the variable construction and statistical analyses used in this research are available in the supplementary files.

## Declaration of interests

None

## Appendix Supplementary materials

Image, application 1

Image, application 2

Image, application 2

Image, application 2

Image, application 2

Image, application 2
