# Supplementary material for: Home modifications and disability outcomes: A longitudinal study of older adults living in England
Source: Lancet Reg Health Eur. 2022 May 4;18:100397. doi: 10.1016/j.lanepe.2022.100397 (PMC9257645; doi:10.1016/j.lanepe.2022.100397)
Supplement: Supplementary file 1 [file mmc1.docx]

**Supplementary Table S1: Derivation of analytical sample used in the main analysis**

| **wave** | **all ELSA waves** | **eligible waves** | **no missing fall** | **no missing moved home** | **no missing wealth** | **no missing CES-D** | **analytical sample** | **with longitudinal weights** |
| --- | --- | --- | --- | --- | --- | --- | --- | --- |
| 1 | 12,099 | 12,099 | 6919 | 5253 | 5183 | 5139 | 5133 | 1250 |
| 2 | 9,432 | 9,432 | 6143 | 4949 | 4869 | 4840 | 4835 | 1641 |
| 3 | 9,771 | .. | .. | .. | .. | .. | .. | .. |
| 4 | 11,050 | .. | .. | .. | .. | .. | .. | .. |
| 5 | 10,274 | 10,274 | 7143 | 6327 | 5961 | 5920 | 5904 | 2676 |
| 6 | 10,601 | 10,601 | 7236 | 6186 | 5715 | 5693 | 5684 | 2887 |
| 7 | 9,666 | 9,666 | 6958 | 5966 | 5432 | 5404 | 5399 | 2867 |
| 8 | 8,445 | 8,445 | 6712 | 5776 | 5202 | 5184 | 5171 | 2864 |
| 9 | 8,736 | .. | .. | .. | .. | .. | .. | .. |
| total observations | 90,074 | 60,517 | 41111 | 34457 | 32362 | 32180 | 32126 | 14185 |
| % reduction | .. | 32·8% | 32·1% | 16·2% | 6·1% | 0·6% | 0·2% | 55·8% |

Abbreviations

CES-D: Center for Epidemiologic Studies-Depression Scale
