# Supplementary material for: Home modifications and disability outcomes: A longitudinal study of older adults living in England
Source: Lancet Reg Health Eur. 2022 May 4;18:100397. doi: 10.1016/j.lanepe.2022.100397 (PMC9257645; doi:10.1016/j.lanepe.2022.100397)
Supplement: Supplementary file 2 [file mmc2.docx]

**Supplementary Table S2: Description of all the variables analysed in the models**

**Outcome variables**

Falls: Any falls in the last two years: no (0) yes (1).

Pain: Often troubled with pain: no (0) yes (1).

Health: Excellent/very good (0) vs good/fair/poor (1).

Social activities: currently participating in paid work/self-employment/voluntary work/education/caring/looking after the home or family: no (0) yes (1).

Moved home by the next wave of ELSA: no (0) yes (1).

**Exposure variables**

External housing modifications: respondent’s home had any of these features: widened doorways, ramps, automatic doors, parking and lift: none (0) one or more (1).

Internal housing modifications: respondent’s home had any of these features: rails, bathroom modifications, kitchen modifications, chair lift: none (0) one or more (1).

Mobility impairment (range 0-10): if the respondent had long term difficulty walking 100 yards/sitting for about two hours/getting up from a chair after sitting for long periods/climbing one flight of stairs without resting/stooping, kneeling, or crouching/reaching or extending your arms above shoulder level/pulling or pushing large objects like a living room chair /lifting or carrying weights over 10 pounds, like a heavy bag/picking up a 5p coin from a table.

**Covariates**

Chronic health conditions (range 0-14): self-reports of the following- high blood pressure, diabetes, cancer, lung disease, heart conditions, stroke, psychological problems, arthritis, cataract, Parkinson, osteoporosis, Alzheimer’s, dementia, and memory related problems.

Sight problems: self-report if eyesight was excellent/very good (0) or good/fair/poor/blind (1).

Hearing problems: self-report if hearing was excellent/very good (0) or /good/fair/poor (1).

Depressive symptoms: 8-item CES-D questionnaire: none (0-2) depressive symptoms (3 or more).

Functional difficulties: 6 item Activities of Daily Living (ADL) questions (range: 0-6).

Single person household: no (0) yes (1).

Coupled relationship: no (0) yes (1).

Household wealth quintiles (range 1-5).

Moderate exercise frequency (range 1-4): more than once/week, once/week, one-three times/month or hardly/never.
