# Supplementary material for: Home modifications and disability outcomes: A longitudinal study of older adults living in England
Source: Lancet Reg Health Eur. 2022 May 4;18:100397. doi: 10.1016/j.lanepe.2022.100397 (PMC9257645; doi:10.1016/j.lanepe.2022.100397)
Supplement: Supplementary file 3 [file mmc3.docx]

Supplementary Table S3: Percentage of disability outcome observations (falls, poor health, pain, no social activities and moved home) and key covariates by exposure variables (mobility impairments and external/internal housing modifications): ELSA analytical sample with longitudinal weights applied (N=14,185)

|  | **No mobility impairments** | | **1+ mobility impairments** | | **No mobility impairments** | | **1+ mobility impairments** | |
| --- | --- | --- | --- | --- | --- | --- | --- | --- |
|  | No ext mods | External mods | No ext mods | External mods | No int mods | Internal mods | No int mods | Internal mods |
| Falls | 18.6% | 21.2% | 33.0% | 35.9% | 18.7% | 21.4% | 30.4% | 40.3% |
| Poor health | 8.0% | 7.9% | 37.9% | 39.4% | 7.8% | 9.7% | 32.0% | 51.0% |
| Pain | 18.5% | 19.3% | 58.1% | 62.8% | 18.4% | 20.1% | 55.1% | 67.3% |
| No social activities | 29.3% | 23.0% | 38.2% | 36.8% | 28.5% | 27.0% | 34.7% | 44.8% |
| Moved home | 4.8% | 4.5% | 4.6% | 5.7% | 4.9% | 4.0% | 4.9% | 4.7% |
| 1+ ADL | 3.8% | 3.1% | 40.8% | 47.0% | 3.3% | 6.0% | 33.0% | 60.7% |
| 1+ health condition | 74.7% | 78.4% | 91.5% | 94.9% | 74.6% | 80.0% | 90.4% | 95.8% |
| Poor sight | 43.8% | 42.8% | 59.2% | 61.7% | 43.1% | 46.8% | 56.1% | 67.1% |
| Poor hearing | 52.6% | 51.8% | 63.6% | 64.7% | 52.5% | 52.8% | 62.1% | 67.4% |
| Dep sympt | 9.5% | 7.9% | 24.5% | 27.1% | 9.1% | 10.6% | 21.7% | 31.9% |
| Single household | 21.4% | 20.6% | 27.8% | 30.8% | 20.8% | 24.1% | 25.0% | 35.6% |
| Couple Relationship | 71.4% | 74.9% | 64.4% | 63.3% | 72.5% | 68.5% | 67.8% | 56.7% |
| No moderate activities | 6.2% | 3.9% | 23.7% | 29.3% | 5.7% | 6.7% | 18.1% | 38.9% |
| Poorest quintile | 10.4% | 8.9% | 19.5% | 22.6% | 9.0% | 18.4% | 16.1% | 28.5% |
| Wealthiest quintile | 27.8% | 38.7% | 15.6% | 19.1% | 30.1% | 25.4% | 19.3% | 10.1% |

Abbreviations:

Ext: external; Int: internal; Mods: modification; ADL: Activities of Daily Living; Dep sympt: Depressive symptoms
