# Supplementary material for: Home modifications and disability outcomes: A longitudinal study of older adults living in England
Source: Lancet Reg Health Eur. 2022 May 4;18:100397. doi: 10.1016/j.lanepe.2022.100397 (PMC9257645; doi:10.1016/j.lanepe.2022.100397)
Supplement: Supplementary file 4 [file mmc4.docx]

**Supplementary Table S4: Coefficients (standard errors) from two way fixed effects models of disability outcomes with mobility impairments, external housing modifications, their interaction and risk factors for disability**

|  | **Fall** | **Pain** | **Poor health** | **No social act** | **Moved home** |
| --- | --- | --- | --- | --- | --- |
| **n observations** | *32,126* | *32,126* | *32,126* | *32,126* | *32,126* |
| **n individuals** | *10,459* | *10,459* | *10,459* | *10,459* | *10,459* |
| % transitions 0 to 1 | 22.2% (n=15,268) | 21.8% (n=12,693) | 13.9% (n=16,119) | 25.1% (n=14,357) | 4.5% (n=20,356) |
| % transitions 1 to 0 | 51.6% (n=6,002) | 29.0% (n=8,577) | 30.1% (n=5,151) | 35.9% (n=6,913) | 88.7% (n=914) |
| ***Mobility imp*** *(ref: no imp)* | **0·016 (0·003)** | **0·054 (0·002)** | **0·029 (0·002)** | -0·001 (0·002) | 0·001 (0·001) |
| ***External mod*** *(ref: no mod)* | **0·033 (0·011)** | **0·025 (0·01)** | 0·002 (0·008) | **-0·063 (0·01)** | **-0·035 (0·006)** |
| ***Interaction (****ref: no imp & no mod)* | |  |  |  |  |
| Mob imp & ext mod | **-0·010 (0·003)** | **-0·008 (0·002)** | **-0·005 (0·002)** | **0·006 (0·003)** | 0·003 (0·002) |
| ***Fall*** *(ref: no falls)* | NA | 0·011 (0·006) | **0·017 (0·005)** | -0·002 (0·007) | **-0·007 (0·004)** |
| ***Pain*** *(ref: no pain)* | 0·014 (0·008) |  | **0·040 (0·006)** | 0·002 (0·008) | 0·004 (0·004) |
| ***Poor health*** *(ref: good health)* | **0·030 (0·009)** | **0·055 (0·009)** |  | **0·036 (0·009)** | 0·005 (0·005) |
| ***No Social Activity*** *(ref: some activity)* | -0·002 (0·007) | 0·001 (0·006) | **0·022 (0·006)** | NA | **0·008 (0·004)** |
| ***Moved home*** *(ref: same home)* | -0·025 (0·013) | 0·012 (0·012) | 0·010 (0·010) | **0·030 (0·014)** | NA |
| ***ADL****diff (range: 0-6)* | **0·015 (0·005)** | **0·012 (0·004)** | **0·020 (0·005)** | **0·017 (0·005)** | -0·0005 (0·003) |
| ***Health condition*** *(range: 0-10)* | <0·001 (0·005) | **0·012 (0·005)** | **0·043 (0·004)** | 0·006 (0·005) | -0·002 (0·003) |
| ***Sight problems*** *(ref: excellent)* | |  |  |  |  |
| very good | 0·002 (0·009) | 0·004 (0·008) | -0·010 (0·007) | 0·001 (0·009) | -0·001 (0·005) |
| good to poor/blind | 0·009 (0·010) | 0·010 (0·009) | **0·021 (0·008)** | 0·013 (0·01) | 0·003 (0·005) |
| ***Hearing problems*** *(ref: excellent)* | |  |  |  |  |
| very good | -0·005 (0·010) | **0·005 (0·009)** | 0·008 (0·007) | 0·014 (0·009) | -0·002 (0·005) |
| good to poor | -0·001 (0·011) | 0·001 (0·010) | **0·025 (0·008)** | 0·004 (0·011) | 0·0003 (0·006) |
| ***Dep sympt*** *(ref: not depressed)* | **0·025 (0·009)** | **0·025 (0·008)** | **0·066 (0·008)** | **0·020 (0·009)** | **0·014 (0·005)** |
| ***Moderate act*** *(ref: > once/week)* | |  |  |  |  |
| once/week | **-0·02 (0·008)** | 0·002 (0·008) | 0·009 (0·007) | 0·002 (0·008) | -0·002 (0·004) |
| 1-3 times/month | -0·008 (0·012) | **0·027 (0·011)** | **0·044 (0·010)** | 0·009 (0·012) | 0·01 (0·006) |
| Hardly/never | -0·015 (0·010) | 0·003 (0·009) | **0·059 (0·009)** | **0·118 (0·011)** | **0·016 (0·006)** |
| ***Single hh*** *(ref: living with others)* | 0·013 (0·020) | **-0·019 (0·017)** | **-0·031 (0·016)** | **0·094 (0·020)** | 0·010 (0·012) |
| ***Coupled*** *(ref: not in relationship)* | |  |  |  |  |
| In relationship | -0·031 (0·020) | **0·082 (0·018)** | 0·004 (0·015) | -0·009 (0·02) | **-0·024 (0·011)** |
| ***Wealth quintiles*** *(ref: poorest)* | |  |  |  |  |
| Quintile 2 | -0·009 (0·021) | -0·007 (0·02) | **0·036 (0·018)** | **-0·040 (0·021)** | **0·033 (0·015)** |
| Quintile 3 | -0·003 (0·023) | <0.001 (0·022) | 0·026 (0·020) | **-0·053 (0·023)** | **0·035 (0·015)** |
| Quintile 4 | -0·020 (0·024) | -0·011 (0·023) | 0·015 (0·021) | **-0·054 (0·025)** | **0·045 (0·016)** |
| Richest quintile | **-0·054 (0·026)** | -0·002 (0·024) | 0·015 (0·022) | **-0·055 (0·027)** | **0·046 (0·017)** |
| ***Wave*** *(ref: w1)* |  |  |  |  |  |
| w2 | **0·024 (0·009)** | **-0·020 (0·008)** | **0·016 (0·007)** | **-0·022 (0·009)** | 0·003 (0·004) |
| w5 | 0·008 (0·011) | -0·003 (0·010) | **0·022 (0·009)** | **0·095 (0·012)** | 0·001 (0·006) |
| w6 | 0·016 (0·012) | 0·016 (0·011) | **0·043 (0·010)** | **0·143 (0·013)** | 0·009 (0·006) |
| w7 | 0·006 (0·013) | 0·007 (0·012) | **0·039 (0·010)** | **0·179 (0·014)** | **0·021 (0·007)** |
| w8 | 0·024 (0·015) | 0·002 (0·013) | **0·035 (0·011)** | **0·218 (0·015)** | 0·010 (0·007) |
| ***Intercept*** | **0·259 (0·029)** | **0·190 (0·026)** | -0·007 (0·023) | **0·211 (0·03)** | 0·015 (0·018) |
| **R-sq:** |  |  |  |  |  |
| within | 0·009 | 0·057 | 0·08 | 0·07 | 0·007 |
| between | 0·108 | 0·362 | 0·43 | 0·04 | 0·000 |
| overall | 0·058 | 0·264 | 0·33 | 0·06 | 0·001 |

**Bold** coefficients denote statistical significance at p<0.05

Mob: mobility; Imp: impairment; Ext: external; Int: internal; Mods: modification; Act: activities; ADL diff: Activities of Daily Living difficulties; Single hh: Single household; Dep sympt: Depressive symptoms; w: wave
