# Supplementary material for: Home modifications and disability outcomes: A longitudinal study of older adults living in England
Source: Lancet Reg Health Eur. 2022 May 4;18:100397. doi: 10.1016/j.lanepe.2022.100397 (PMC9257645; doi:10.1016/j.lanepe.2022.100397)
Supplement: Supplementary file 5 [file mmc5.docx]

**Supplementary Table S5: Coefficients (standard errors) from two way fixed effects models of disability outcomes with mobility impairments, internal housing modifications, their interaction and risk factors for disability**

|  | ***Fall*** | ***Pain*** | ***Poor health*** | ***No social activities*** | ***Moved home*** |
| --- | --- | --- | --- | --- | --- |
| **n observations** | *32,126* | *32,126* | *32,126* | *32,126* | *32,126* |
| **n individuals** | *10,459* | *10,459* | *10,459* | *10,459* | *10,459* |
| % transitions 0 to 1 | 22·2% (n=15,268) | 21·8% (n=12,693) | 13·9% (n=16,119) | 25·1% (n=14,357) | 4·5% (n=20,356) |
| % transitions 1 to 0 | 51·6% (n=6,002) | 29·0% (n=8,577) | 30·1% (n=5,151) | 35·9% (n=6,913) | 88·7% (n=914) |
| ***Mobility imp*** *(ref: no imp)* | **0·014 (0·003)** | **0·058 (0·003)** | **0·029 (0·002)** | -0·002 (0·003) | -0·0003 (0·001) |
| ***Internal mod*** *(ref: no mod)* | 0·015 (0·012) | **0·027 (0·011)** | **0·024 (0·009)** | **-0·046 (0·012)** | **-0·022 (0·006)** |
| ***Interaction (****ref: no imp & no mod)* | |  |  |  |  |
| Mob imp & int mod | -0·001 (0·003) | **-0·013 (0·002)** | **-0·004 (0·002)** | **0·008 (0·003)** | **0·005 (0·002)** |
| ***Fall*** *(ref: no falls)* | NA | 0·012 (0·006) | **0·017 (0·005)** | -0·002 (0·007) | -0·007 (0·004) |
| ***Pain*** *(ref: no pain)* | 0·014 (0·008) | NA | **0·040 (0·006)** | 0·002 (0·008) | 0·004 (0·004) |
| ***Poor health*** *(ref: good health)* | **0·030 (0·009)** | **0·055 (0·009)** | NA | **0·037 (0·009)** | 0·005 (0·005) |
| ***No Social Activity*** *(ref: some activity)* | -0·002 (0·007) | 0·001 (0·006) | **0·022 (0·006)** |  | **0·009 (0·004)** |
| ***Moved home*** *(ref: same home)* | **-0·026 (0·013)** | 0·012 (0·012) | 0·011 (0·010) | **0·032 (0·014)** |  |
| ***ADL****diff (range: 0-6)* | **0·014 (0·005)** | **0·014 (0·004)** | **0·020 (0·005)** | **0·017 (0·005)** | -0·001 (0·003) |
| ***Health condition*** *(range: 0-10)* | -0·001 (0·005) | **0·012 (0·005)** | **0·043 (0·004)** | 0·006 (0·005) | -0·002 (0·003) |
| ***Sight problems*** *(ref: excellent)* | |  |  |  |  |
| very good | 0·002 (0·009) | 0·004 (0·008) | -0·010 (0·007) | **0·001 (0·009)** | **-0·001 (0·005)** |
| good to poor/blind | 0·009 (0·010) | 0·01 (0·009) | **0·022 (0·008)** | 0·013 (0·010) | 0·003 (0·005) |
| ***Hearing problems*** *(ref: excellent)* | |  |  |  |  |
| very good | -0·005 (0·010) | 0·004 (0·009) | 0·008 (0·007) | 0·015 (0·009) | -0·002 (0·005) |
| good to poor | -0·002 (0·011) | 0·001 (0·010) | **0·024 (0·008)** | 0·005 (0·011) | 0·001 (0·006) |
| ***Dep sympt*** *(ref: not depressed)* | **0·025 (0·009)** | **0·026 (0·008)** | **0·066 (0·008)** | **0·019 (0·009)** | **0·014 (0·005)** |
| ***Moderate act*** *(ref: > once/week)* | |  |  |  |  |
| once/week | **-0·02 (0·008)** | 0·002 (0·008) | 0·010 (0·007) | 0·003 (0·008) | -0·002 (0·004) |
| 1-3 times/month | -0·008 (0·012) | **0·026 (0·011)** | **0·044 (0·010)** | 0·009 (0·012) | 0·010 (0·006) |
| Hardly/never | -0·016 (0·010) | 0·003 (0·009) | **0·059 (0·009)** | **0·119 (0·011)** | **0·017 (0·006)** |
| ***Single hh*** *(ref: living with others)* | 0·013 (0·02) | **-0·016 (0·017)** | -0·030 (0·016) | **0·093 (0·020)** | 0·009 (0·012) |
| ***Coupled*** *(ref: not in relationship)* | |  |  |  |  |
| In relationship | -0·031 (0·02) | **0·083 (0·018)** | 0·005 (0·015) | -0·010 (0·020) | **-0·025 (0·011)** |
| ***Wealth quintiles*** *(ref: poorest)* | |  |  |  |  |
| Quintile 2 | -0·007 (0·021) | -0·008 (0·020) | **0·037 (0·018)** | **-0·042 (0·021)** | **0·032 (0·015)** |
| Quintile 3 | -0·002 (0·023) | -0·001 (0·022) | 0·026 (0·020) | **-0·055 (0·023)** | **0·035 (0·015)** |
| Quintile 4 | -0·018 (0·024) | -0·011 (0·023) | 0·016 (0·021) | **-0·058 (0·025)** | **0·043 (0·016)** |
| Richest quintile | **-0·053 (0·026)** | -0·002 (0·024) | 0·016 (0·022) | **-0·058 (0·027)** | **0·045 (0·017)** |
| ***Wave*** *(ref: w1)* |  |  |  |  |  |
| w2 | **0·024 (0·009)** | **-0·020 (0·008)** | **0·015 (0·007)** | **-0·021 (0·009)** | 0·003 (0·004) |
| w5 | 0·007 (0·011) | -0·002 (0·010) | **0·020 (0·009)** | **0·097 (0·012)** | 0·002 (0·006) |
| w6 | 0·017 (0·012) | 0·018 (0·011) | **0·040 (0·010)** | **0·140 (0·013)** | 0·006 (0·006) |
| w7 | 0·007 (0·013) | 0·010 (0·012) | **0·036 (0·010)** | **0·174 (0·014)** | **0·016 (0·007)** |
| w8 | 0·025 (0·014) | 0·006 (0·013) | **0·031 (0·011)** | **0·212 (0·015)** | 0·005 (0·007) |
| ***Intercept*** | **0·261 (0·029)** | **0·185 (0·026)** | -0·010 (0·024) | 0·215 (0·03) | 0·017 (0·018) |
| **R-sq:** |  |  |  |  |  |
| overall | 0·009 | 0·058 | 0·079 | 0·066 | 0·006 |
| between | 0·110 | 0·368 | 0·429 | 0·033 | <0·0001 |
| overall | 0·059 | 0·268 | 0·327 | 0·055 | 0·001 |

**Bold** coefficients denote statistical significance at p<0.05

Mob: mobility; Imp: impairment; Ext: external; Int: internal; Mods: modification; Act: activities; ADL diff: Activities of Daily Living difficulties;; Single hh: Single household; Dep sympt: Depressive symptoms; w: wave
