# Supplementary material for: Home modifications and disability outcomes: A longitudinal study of older adults living in England
Source: Lancet Reg Health Eur. 2022 May 4;18:100397. doi: 10.1016/j.lanepe.2022.100397 (PMC9257645; doi:10.1016/j.lanepe.2022.100397)
Supplement: Supplementary file 6 [file mmc6.docx]

**Supplementary Table S6: Coefficients (standard errors) from sensitivity analyses comparing coefficients from the main analytical sample (linear regression models), weighted sample (linear regression models) and main analytical sample (logistic regression models)**

S6a: Models including **external** housing modifications as an independent variable

|  | Main sample (reg) | Weighted sample (reg) | Main sample (logit) | Main sample (weighted logit) |
| --- | --- | --- | --- | --- |
| **Fall** |  |  |  |  |
| Mobility imp (ref: no) | **0·016 (0·003)** | **0·017 (0·004)** | **0·090 (0·015)** | **0.100 (0.024)** |
| External mod (ref: no mod) | **0·033 (0·011)** | **0·026 (0·015)** | **0·228 (0·072)** | 0.179 (0.100) |
| Interaction (ref: no imp & no mod) | **-0·010 (0·003)** | -0·008 (0·005) | **-0·071 (0·019)** | -0.053 (0.029) |
| **Pain** |  |  |  |  |
| Mobility imp (ref: no) | **0·054 (0·002)** | **0·034 (0·004)** | **0·373 (0·018)** | **0.439 (0.028)** |
| External mod (ref: no mod) | **0·025 (0·01)** | 0·012 (0·014) | **0·198 (0·083)** | **0.215 (0.104)** |
| Interaction (ref: no imp & no mod) | **-0·008 (0·002)** | -0·005 (0·040) | **-0·068 (0·026)** | **-0.104 (0.040)** |
| **Poor health** |  |  |  |  |
| Mobility imp (ref: no) | **0·029 (0·002)** | **0·034 (0·004)** | **0·201 (0·018)** | **0.270 (0.028)** |
| External mod (ref: no mod) | 0·002 (0·008) | <0·001 (0·010) | 0·115 (0·112) | 0.107 (0.152) |
| Interaction (ref: no imp & no mod) | **-0·005 (0·002)** | -0·007 (0·004) | **-0·07 (0·027)** | **-0.091 (0.039)** |
| **No Social Activities** |  |  |  |  |
| Mobility imp (ref: no) | -0·001 (0·002) | -0·004 (0·004) | -0·006 (0·015) | -0.008 (0.023) |
| External mod (ref: no mod) | **-0·063 (0·01)** | **-0·050 (0·020)** | **-0·424 (0·079)** | **-0.316 (0.106)** |
| Interaction (ref: no imp & no mod) | **0·006 (0·003)** | **0·010 (0·005)** | **0·042 (0·020)** | 0.008 (0.028) |
| **Moved home** |  |  |  |  |
| Mobility imp (ref: no) | 0·001 (0·001) | <0·001(0·002) | 0·009 (0·03) | 0.036 (0.040) |
| External mod (ref: no mod) | **-0·035 (0·006)** | -0·015 (0·009) | **-0·434 (0·129)** | **-0.487 (0.185)** |
| Interaction (ref: no imp & no mod) | 0·003 (0·002) | 0·004 (0·002) | 0·039 (0·033) | 0.005 (0.051) |

S6b: Models including **internal** housing modifications as an independent variable

|  | Main sample (reg) | Weighted sample (reg) | Main sample (logit) | Main sample (weighted logit) |
| --- | --- | --- | --- | --- |
| **Fall** |  |  |  |  |
| Mobility imp (ref: no) | **0·014 (0·003)** | **0·017 (0·004)** | **0·079 (0·016)** | **0.095 (0.025)** |
| Internal mod (ref: no mod) | 0·015 (0·012) | 0·004 (0·017) | 0·093 (0·074) | 0.018 (0.112) |
| Interaction (ref: no imp & no mod) | -0·001 (0·003) | -0·002 (0·005) | -0·009 (0·018) | -0.014 (0.029) |
| **Pain** |  |  |  |  |
| Mobility imp (ref: no) | **0·058 (0·003)** | **0·060 (0·004)** | **0·393 (0·019)** | **0.441 (0.029)** |
| Internal mod (ref: no mod) | **0·027 (0·011)** | 0·008 (0·016) | 0·166 (0·087) | -0.036 (0.115) |
| Interaction (ref: no imp & no mod) | **-0·013 (0·002)** | **-0·011 (0·004)** | **-0·090 (0·024)** | -0.056 (0.037) |
| **Poor health** |  |  |  |  |
| Mobility imp (ref: no) | **0·029 (0·002)** | **0·034 (0·004)** | **0·204 (0·02)** | **0.281 (0.029)** |
| Internal mod (ref: no mod) | **0·024 (0·009)** | 0·012 (0·014) | 0·193 (0·106) | 0.200 (0.155) |
| Interaction (ref: no imp & no mod) | **-0·004 (0·002)** | -0·005 (0·004) | -0·047 (0·025) | **-0.081 (0.038)** |
| **No Social Activities** |  |  |  |  |
| Mobility imp (ref: no) | -0·002 (0·003) | -0·004 (0·004) | -0·014 (0·016) | -0.024 (0.024) |
| Internal mod (ref: no mod) | **-0·046 (0·012)** | **-0·051 (0·019)** | **-0·306 (0·077)** | **-0.358 (0.119)** |
| Interaction (ref: no imp & no mod) | **0·008 (0·003)** | **0·009 (0·005)** | **0·044 (0·019)** | 0.055 (0.027) |
| **Moved home** |  |  |  |  |
| Mobility imp (ref: no) | -0·0003 (0·001) | <0·001 (0·002) | -0·007 (0·032) | 0.013 (0.042) |
| Internal mod (ref: no mod) | **-0·022 (0·006)** | -0·015 (0·009) | **-0·31 (0·143)** | **-0.377 (0.207)** |
| Interaction (ref: no imp & no mod) | **0·005 (0·002)** | 0·004 (0·002) | 0·066 (0·035) | 0.07 (0.048) |

**Bold** coefficients denote statistical significance at p<0.05

Mob: mobility; Imp: impairment; Ext: external; Int: internal; Mods: modification
